# From the Au nano-clusters to the nanoparticles on 4H-SiC (0001)

**DOI:** 10.1038/srep13954

**Published:** 2015-09-10

**Authors:** Ming-Yu Li, Quanzhen Zhang, Puran Pandey, Mao Sui, Eun-Soo Kim, Jihoon Lee

**Affiliations:** 1 College of Electronics and Information, Kwangwoon University, Nowon-gu Seoul 139-701, South Korea; 2 Institute of Nanoscale Science and Engineering, University of Arkansas, Fayetteville AR 72701, USA

## Abstract

The control over the configuration, size, and density of Au nanoparticles (NPs) has offered a promising route to control the spatial confinement of electrons and photons, as a result, Au NPs with a various configuration, size and density are witnessed in numerous applications. In this work, we investigate the evolution of self-assembled Au nanostructures on 4H-SiC (0001) by the systematic variation of annealing temperature (AT) with several deposition amount (DA). With the relatively high DAs (8 and 15 nm), depending on the AT variation, the surface morphology drastically evolve in two distinctive phases, i.e. (I) irregular nano-mounds and (II) hexagonal nano-crystals. The thermal energy activates adatoms to aggregate resulting in the formation of self-assembled irregular Au nano-mounds based on diffusion limited agglomeration at comparatively low annealing temperature, which is also accompanied with the formations of hillocks and granules due to the dewetting of Au films and surface reordering. At high temperature, hexagonal Au nano-crystals form with facets along {111} and {100} likely due to anisotropic distribution of surface energy induced by the increased volume of NPs. With the small DA (3 nm), only dome shaped Au NPs are fabricated along with the variation of AT from low to elevated temperature.

Due to its wide band-gap, high current tolerance and high electron mobility, SiC is widely used in high power devices^1^,^2^,^3^,^4^,^5^. α-SiC (3C-SiC) show zinc blende structure while β-SiC (4 H- and 6H-SiC) consists of wurtzite. Among the poly-types, the 4H-SiC possesses the highest band-gap of 3.26 eV and the 6H shows 3.02 eV while 3C shows much lower value at 2.39 eV^6^. Recently, SiC are extensively applied for the fabrication of the high quality epitaxial graphene layers due to their thermal decomposition with a preferential sublimation of Si^7^, and the C-terminated surface generally requires lower temperature than Si-terminated surface to grow a graphene film with an identical thickness as a result of a more rapid sublimation^8^. On the other hand, owing to the localized surface plasmon resonance and large surface to volume ratio, Au NPs has received extensive research attentions for the optical^9^,^10^,^11^, electric^12^ and biological^13^ applications. The variation of shape, size and density of Au NPs can provide an proficient way to optimize the performance of the corresponding devices such as enhanced light absorption in the solar cells^14^, the performance of the localized surface plasmon resonance transducers by determining the surface plasmon decay and refractive index sensitivity^15^, controlling the memory window of FETs^12^. Also, the Au NPs with a remarkable catalytic capacity^16^ can serve as nucleation sites for NWs by absorbing the vaporized target materials based on the vapor-liquid-solid growth mechanism^17^ and the diameter and length^18^, density^19^,^20^, direction^21^, and shape^22^ of the NWs can be inherently determined by that of the Au NPs. Recently, Au nanoparticles (NPs) have been applied to control the Schottky barrier height with a variation of its size on 4H-SiC^23^,^24^,^25^. Au NPs have a potential of being applied in the various applications, however, the research on 4H-SiC is still relatively deficient and therefore, in this work we systematically investigate the controlled evolution of the various self-assembled Au nanostructures on 4H-SiC (0001) by the variation of annealing temperature (AT) with various deposition amounts (DAs). As shown in Fig. 1, depending on the DA, various nanostructures are fabricated, and evolve along with the increased AT. For example, with the 15 nm DA, Au nanostructures undergo drastic evolution in configurations with two distinctive phases: (I) irregular Au nano-mounds and (II) hexagonal Au nano-crystals. Phase I: With the thermal energy supplied, Au adatoms can gradually diffuse and aggregated at the pinholes perforated by the voids to form the irregular Au nano-mounds along with the formation of the hillocks and granules at relatively low annealing temperature which can be described with the diffusion limited agglomeration model^26^, as shown in Fig. 1(a),(a-1) and (a-2). Phase II: With the AT increase, all the Au structures gradually develop into the hexagonal nano-crystals owing to the enhanced surface diffusion, and the truncated facets formed to minimize the anisotropic surface energy, as shown with Fig. 1(b),(b-1). On the other hand, at relatively low DA (3 nm), the agglomeration process immediately proceed to the formation of dome-shaped Au NPs based on the Volmer-Weber growth model^27^,^28^, and the Au NPs evolve with the increased size at the expense of the small Au NPs as a function of the AT.

## Results and Discussion

**Figure 1:**
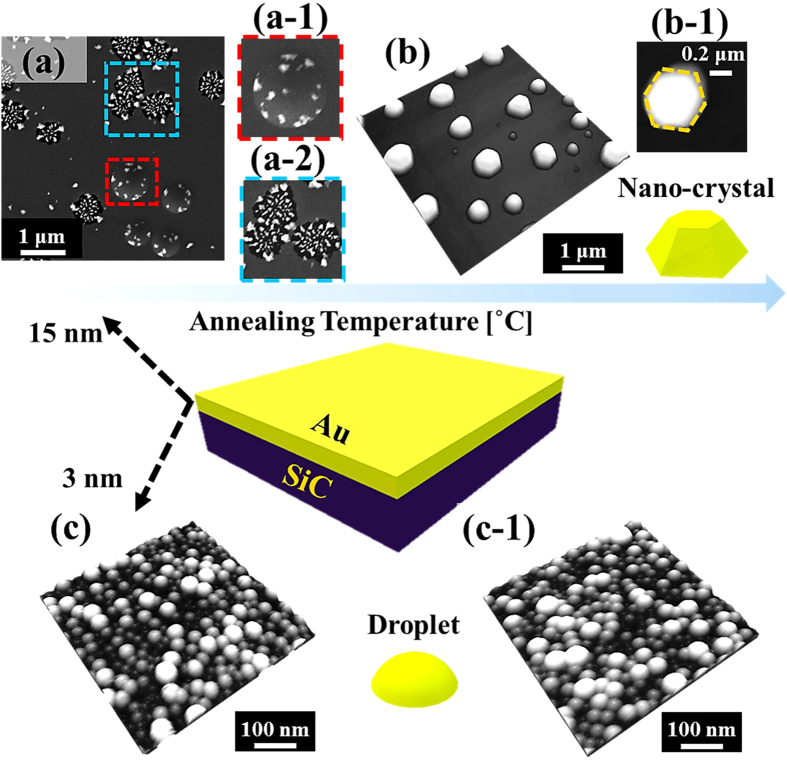
Illustration of the fabrication of self-assembled Au nanostructures on 4H-SiC (0001) by the control of annealing temperature (AT) with various Au deposition amounts (DAs). (**a**) Scanning electron microscopy (SEM) image (5 × 5 μm^2^) of a sample annealed at 600 °C with the 15 nm DA. (**a-1**)–(**a-2**) SEM images of a hillocks (1.6 × 1.6 μm^2^) and pinholes (1.3 × 1.3 μm^2^). (**b**) Atomic force microscope (AFM) side-view of hexagonal Au nano-crystals at 850 °C (5 × 5 μm^2^). (**b-1**) AFM top-view (1 × 1 μm^2^) of a hexagonal nano-crystal. (**c**,**d**) Dome-shaped Au nano-particles fabricated with a DA of 3 nm at 800 °C and 900 °C. (**c**,**d**) AFM top-views of 500 × 500 nm^2^.

**Figure 2:**
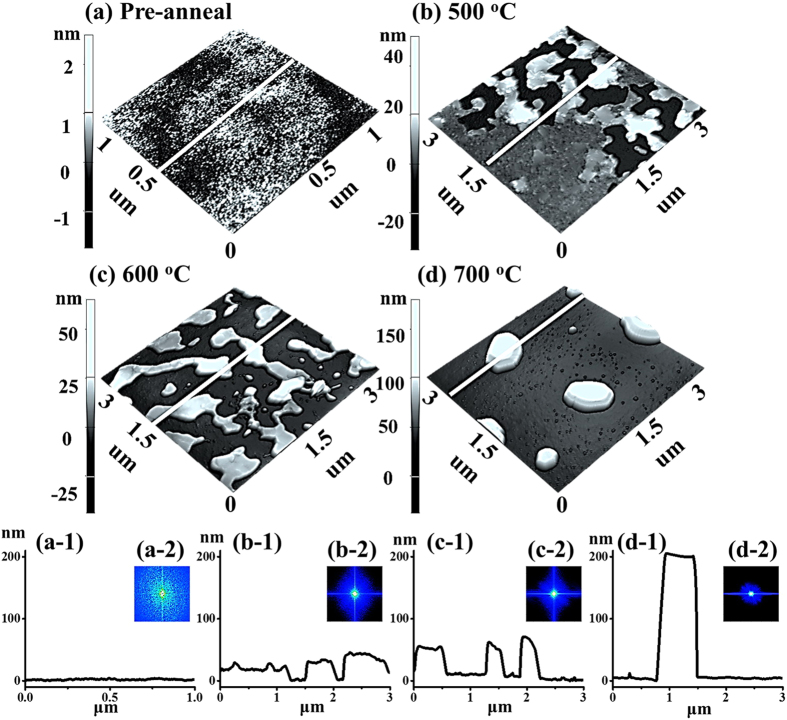
Evolution of the self-assembled Au nano-mounds on 4H-SiC (0001) at various annealing temperatures (AT) between 500 and 700 °C with 8 nm of Au deposition. (**a**,**d**) AFM side-views of 1 × 1 and 3 × 3 μm^2^. (**a-1**)–(**d-1**) Cross sectional line-profiles. (**a-2**)–(**d-2**) Two dimensional (2-D) Fourier filter transform (FFT) power spectra.

Figure 2 shows the fabrication of self-assembled Au nano-mounds on 4H-SiC (0001) by the variation of the annealing temperature (AT) between 500 and 700 °C. Corresponding SEM images are provided in Fig. S4. In general, with the AT variation, the heterogeneous dewetting of Au film gradually occurred as a function of surface energy, resulting in a drastic surface morphology evolution from a continuous Au thin film to isolated irregular Au nano-mounds, which can be described in conjunction with a diffusion limited agglomeration (DLA) model^26^. Initially, since the deposited Au thin film can generally possess a high vacancy concentration^26^,^29^, being providing with the thermal energy, Au adatoms can spontaneously respond to diffuse, which can cause the nucleation of the vacancies, and in turn to form voids at random nucleation sites including highly strained sites and grain boundaries induced by the thermal expansion coefficient mismatch between Au and the SiC. Subsequently, the voids were kept forming with increased vacancy nucleation, and can perforated the Au film to form pinholes. Meanwhile, the agglomeration of Au can be initiated in the pinholes with a radius (*R*_*p*_) bigger than the critical size (*R*_*C*_), which can be expressed as 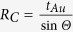
30, where t_Au_ is the thickness of Au layer. Also, the equilibrium contact angle of the Au nano-particle (Θ) can be described with the interfacial energy densities of γ_SiC/Vac_ (between SiC and vacuum), γ_Au/SiC_ (between Au and SiC), and γ_Au/Vac_ (between Au and vacuum) as 
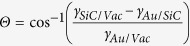
. Namely, the pinholes with an *R*_*p*_ bigger than the *R*_*C*_ can possess a stronger driving force for the agglomeration (dewetting), which can eventually result in the formation of the irregular nano-mounds. Meanwhile, based on the thermodynamic diffusion theory, the *l*_*D*_ can be expressed as 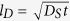, where *t* is residence time of Au adatoms. And the *D*_*s*_ (diffusion coefficient) can be given by 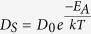
31, where *k* is Boltzmann constant, *D*_*0*_ and *E*_*A*_ (the diffusion barrier) are with certain values under an identical growth condition, therefore, the *l*_*D*_ can be determined by the variation of the *T* (surface temperature). Consequently, an enhanced diffusion length can be expected with more thermal energy supplied, and as a result, when the AT was increased, more pinholes can be formed with a proper size (*R*_*p*_ *>* *R*_*C*_), which can correspondingly enhance the agglomeration initiation. On the other hand, with the increased *l*_*D*_, the connected irregular nano-mounds can have a tendency to expand and separate into isolated ones due to the Rayleigh instability^32^,^33^ as shown with AFM side- and top-views in Fig. 2, S3. In specific, the surface morphology with the 8 nm-thick Au deposition appeared quite smooth with only a few of nanometers of surface modulation, as shown in Fig. 2(a),(a-1). After being annealed at 500 °C, partial formation of the connected nano-mounds occurred in the Au film due to the limited Au adatom diffusion as shown in Fig. 2(b),(b-1). When the AT was increased to 600 and 700 °C, Au adatoms aggregated more compactly, resulting in the shape transition from the connected Au nano-mounds to the isolated ones with a drastic vertical size expansion as shown in Fig. 2(c-1)–(d-1). In addition, the morphology evolution can be clearly observed with the sharp increases in both the root-mean-squared roughness (R_RMS_) and surface area ratio (SAR), suggesting that the significant vertical size increase along with the AT increase, as shown in Fig. S3(e). As summarized in Table SI, the R_RMS_ increased ×47.7 times from ~1 to ~47.7 nm, and the SAR increased from 0.09% to 8.27%, correspondingly. As a result, the drastic changes in the two dimensional (2-D) Fourier filter transform (FFT) power spectra along with the surface morphology evolution also can be similarly witnessed in Fig. 2(a-2)–(d-2). The symmetric bright spot indicating random distributed height drastically shrunk into a small spot caused by the reduction in the height distribution along with the vertical size increase. In brief, during the annealing between 500 and 700 °C the surface morphology underwent a drastic evolution from the flat Au thin film with only few nanometer modulation to the irregular Au nano-mounds with several hundred nanometers in height due to the enhanced surface diffusion as a function of temperature, which also can be equally observed with large-scale scanning electron microscopy (SEM) images in Fig S4. Similar evolution of the irregular Au nano-mounds can be observed on quartz substrate^34^, various GaAs^35^,^36^, sapphire^37^ and soft polymeric substrates^38^.

**Figure 3:**
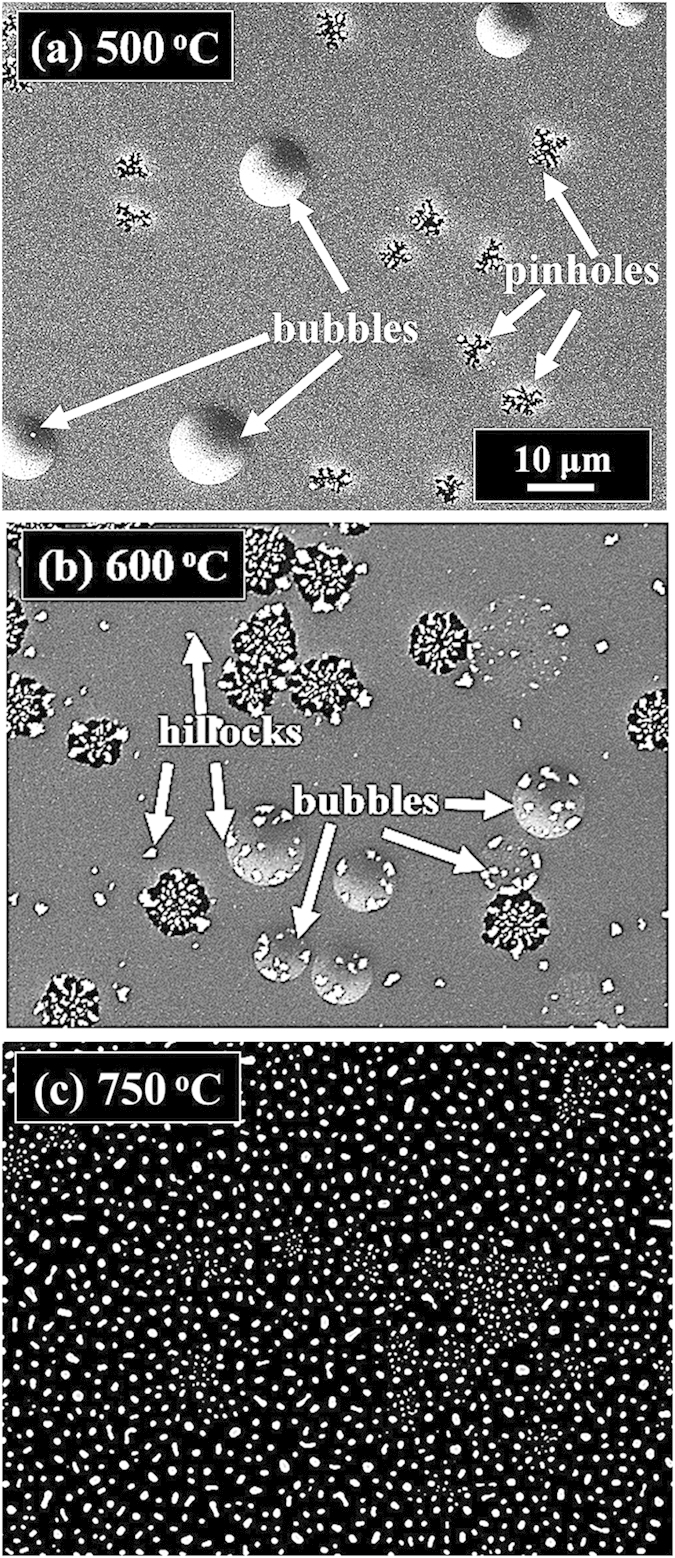
Formation of hillocks, voids, granules and Au nano-mounds with 15 nm Au deposition by annealing between 500–750 °C on 4H-SiC (0001). SEM images are of 100 (x) × 76.67 (y) μm^2^.

**Figure 4:**
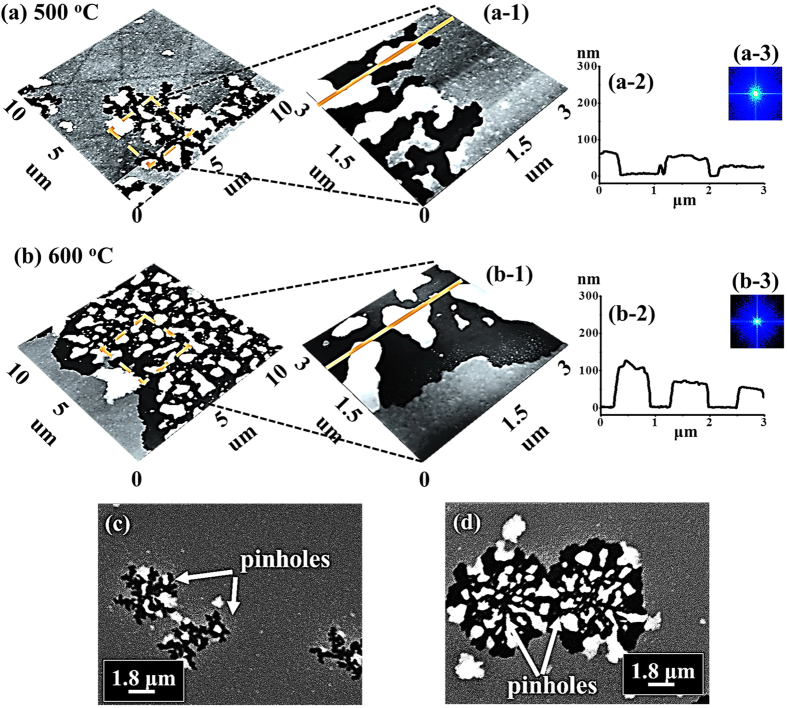
Initial stages of the fabrication of Au nanostructure annealed at 500 and 600 °C with a DA of 15 nm. (**a**,**b**) AFM side-views of 10 × 10 μm^2^. (**a-1**)–(**b-1**) Enlarged AFM side-views of 3 × 3 μm^2^. (**a-2**)–(**b-2**) Cross sectional line-profiles. (**a-3**)–(**b-3**) FFT power spectra. (**c**,**d**) SEM images (18.2 × 13.9 μm^2^) of the samples annealed at 500 °C and 600 °C.

**Figure 5:**
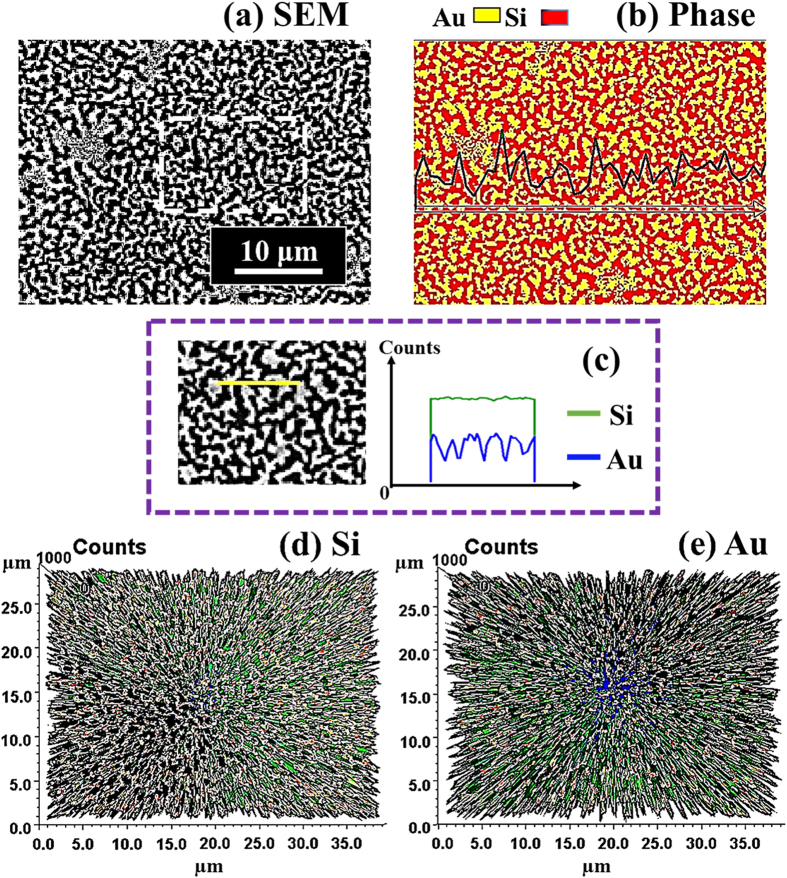
Elemental analysis of self-assembled Au nanostructures on 4H-SiC (0001) by the energy-dispersive X-ray spectroscopy (EDS). (**a**) SEM image of the sample with the 8 nm DA annealed at 600 °C. (**b**) Combined EDS phase map of Au (yellow) and Si (red). (**c**) Line-profiles of element counts of Si (green) and Au (blue) denoted with the yellow line in the SEM image. (**d**,**e**) 3-D top-view phase maps of Si and Au.

Figure 3 shows the evolution of the self-assembled irregular Au nano-mounds with 15 nm DA on 4H-SiC (0001) controlled by the variation of the AT between 500 and 750 °C. The detailed surface morphology changes at the initial stage of the Au thin film agglomeration are presented in Fig. 4 and S5. Similar to the 8 nm Au deposition, Au adatoms gradually aggregated and developed into the isolated irregular nano-mounds with the incremental variation of AT. Meanwhile, the formation of pinholes and granules was simultaneously witnessed during the evolution of Au nano-mounds, as shown with SEM images in Fig. 3. More specifically, as shown in Fig. 3(a), hillocks were formed with diameters of several micrometers or even larger than 10 micrometers at 500 °C possibly because of the thermal expansion of Au films. The hillocks appeared to the prior process of the pinhole formation at increased thermal energy, and the hillocks would eventually evolve into the pinholes with the formation of nano-mounds subsequently, as mentioned. At 600 °C of annealing, with the increased thermal energy, the number of hillocks and pinholes were further increased, as clearly shown in Fig. 3(b). Also, the granules started forming on the hillocks due to the tendency of the Au film to release the compressive stress, which resulted from the higher thermal coefficient of the Au film than the substrate. The hillock formation was also observed with the Au films on Y_2_O_3_-doped ZrO_2_ (YSZ)^39^,^40^. Finally, when the AT was reached 750 °C, all the Au structures agglomerated into the isolated Au nano-mounds with a uniform distribution owing to the enhanced diffusion of Au adatoms as shown in Fig. 3(c). At the stage of the pinholes formation, with the temperature increase, the size of the pinholes noticeably extended because of the more drastic agglomeration, as shown with the AFM side-views, line-profiles and top-views and SEM images in Fig. 4 and S5. Figure 5 shows the elemental analysis of the irregularly connected Au nano-mounds by the energy-dispersive X-ray spectroscopy (EDS). As shown by the combined EDS phase map in Fig. 5(b), the Au (yellow color) and Si phases (red color) were clearly matched the surface morphology shown by the SEM image in Fig 5(a). The Si (green line) can be observed everywhere on the surface, whereas, Au counts (blue line) mainly distributed along the nano-mounds, suggesting the agglomeration occurred with the limited diffusion, as shown by line-profiles indicating by the yellow line in the enlarged image in Fig. 5(c). Similarly, more noticeable counts of Au existed at the area with nano-mounds, and rest area were full of the Si counts, as shown in Fig. 5(d–e).

**Figure 6:**
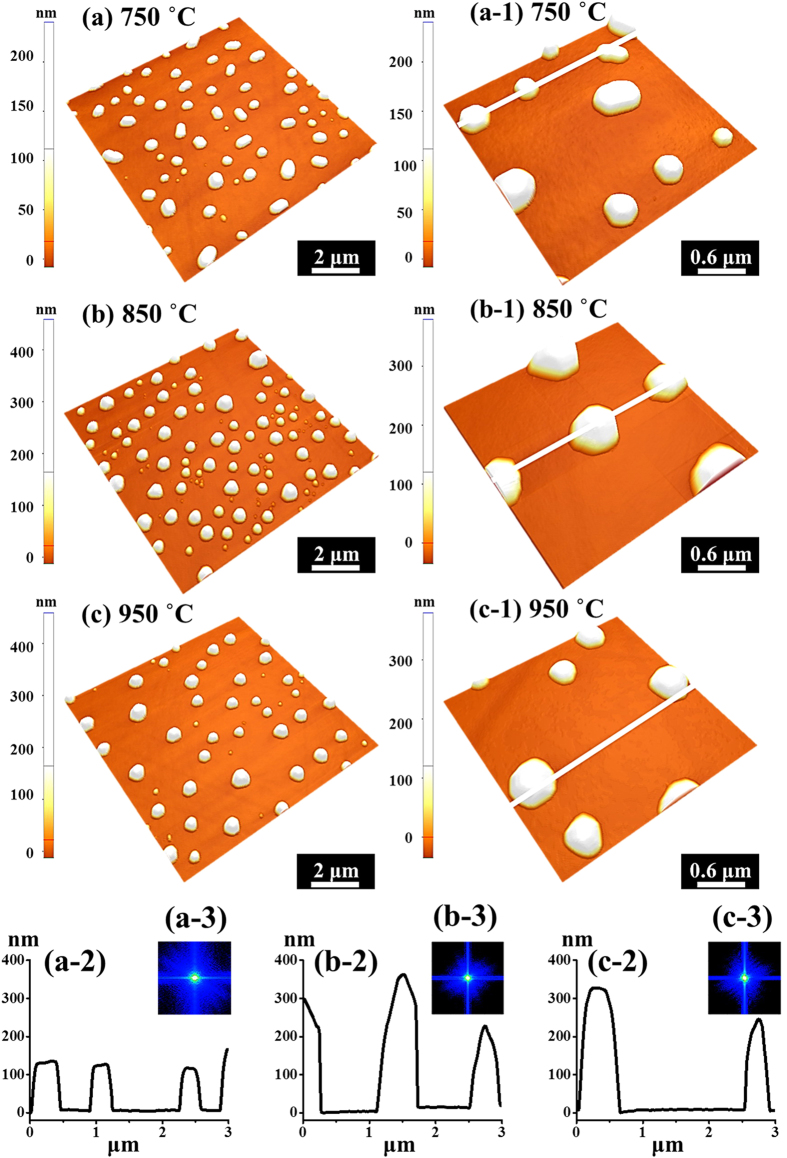
Self-assembled Au nano-crystals fabricated at various ATs between 750 and 950 °C with a DA of 15 nm. (**a**–**c**) AFM side-views of 10 × 10 μm^2^. (**a-1**)–(**c-1**) 3 × 3 μm^2^. (**a-2**)–(**c-2**) Cross sectional line-profiles. (**a-3**)–(**b-3**) FFT power spectra.

**Figure 7:**
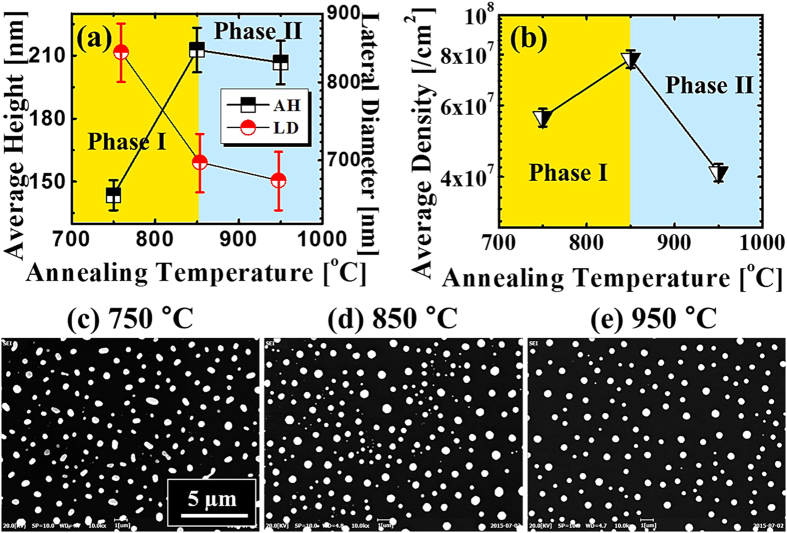
Summary of average height (AH), lateral diameter (LD) and average density (AD) of Au nano-crystals at various ATs between 750 and 950 °C with 15 nm DA. (**a**) Average height (AH) and lateral diameter (LD) at each temperature. (**b**) Average density (AD) at each temperature. Error bars are ±5%. (**c**–**e**) SEM images of 19.4 (x) × 14.6 (y) μm^2^ at each AT.

**Figure 8:**
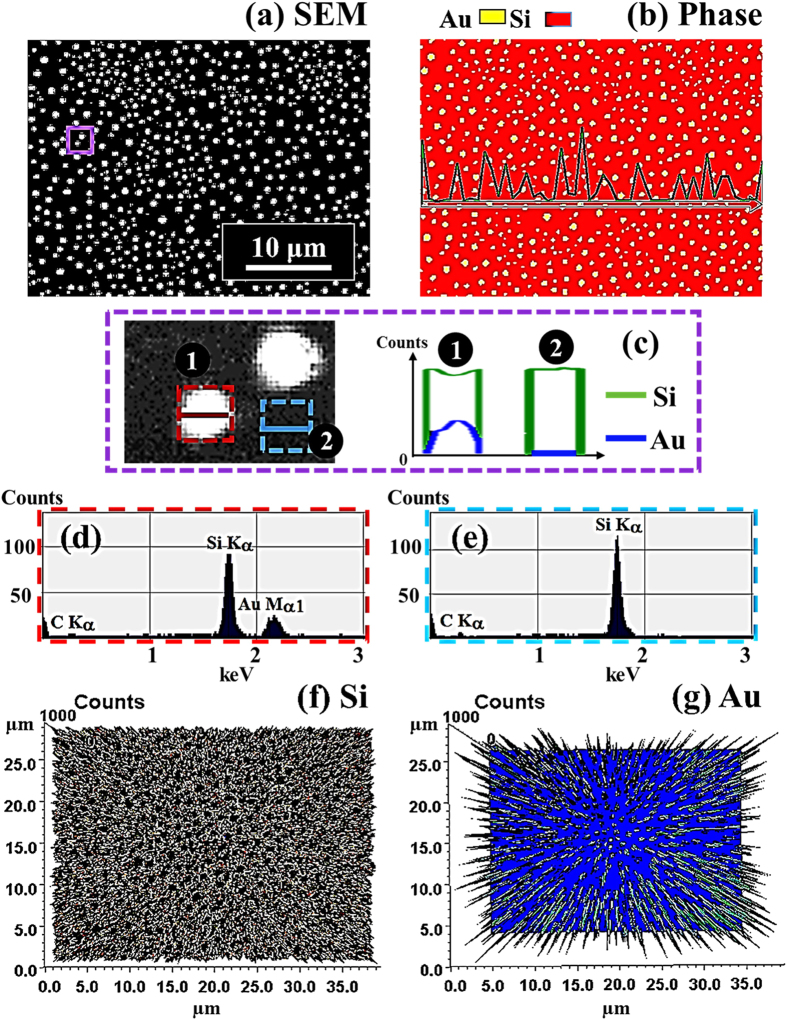
EDS phase maps and spectra of the Au nano-crystals fabricated at 900 °C with the 15 nm DA on 4H-SiC (0001). (**a**) SEM image of 40(x) × 30(y) μm^2^. (**b**) Combined phase map of Au (yellow) and Si (red). (**c**) Line-profiles of element counts of Si (green) and Au (blue). (**d**,**e**) Corresponding EDS spectra from the red box (**d**) and blue (**e**). (**f**,**g**) 3-D top-view phase maps of Si and Au.

Figure 6 shows the transition phase between the Au nano-mounds and Au nano-crystals at a higher AT range between 750 and 950 °C with a DA of 15 nm on 4H-SiC (0001). The corresponding AFM top-views and SEM images are shown in Fig. 7, S6 and S7 respectively. Generally, Au nanostructure fabrication was quite sensitive to the AT, namely, above a certain AT, the hexagonal Au nano-crystals can be successfully synthesized. The equilibrium shape of NPs can be decided by the Wulff construction resulted from the orientation dependence of surface energy^41^,^42^, and thus, with each fixed growth condition the shape of NPs tend to minimize the surface energy within a certain volume. The face-centered-cubic (fcc) materials, such as Au, have a tendency to truncate facets to reduce the surface energy and consequently, with the accumulation of Au adatoms, the anisotropy gradually appears more obviously. Thus, as soon as reaching the critical volume, the facet truncation can happen along each {111} and {100}, finally resulting in the formation of the hexagonal nano-crystals^41^. In more detail, at 750 °C, the agglomeration (Au nano-mounds separation) was still dominant due to the insufficient diffusion, which can be evidenced with some elongated nano-mounds (phase I), as shown in Figs 6(a),(a-1) and 7. Being providing with a sufficient thermal energy at 850 °C, the nano-mounds gradually separated into Au nano-crystals with a noticeable size increase, as shown in Fig. 6(b),(b-1). As mentioned, owing to the anisotropic surface energy, the Au nano-crystals were turned into the hexagonal shape as shown in Fig. 6(a-1) (phase II). When the AT reached 950 °C, the hexagonal nano-crystals can still be fabricated with a slight decrease in both the size and the density, as shown in Fig. 6(c),(c-1). The size reduction at a higher surface temperature associated with the density decrease is against the general trend of surface diffusion, and this can be possibly due to the enhanced evaporation of nanoscale Au as a function of AT, which can similarly witnessed on Si^43^, MgO, SrTiO_3_, and Al_2_O_3_^44^. To specify the size and density evolution, the average height (AH), lateral diameter (LD) and average density (AD) are summarized in Fig. 7(a,b) and Table SII, which can be divided into two phases: phase I between 750 and 850 °C and phase II above 850 °C. The size and density evolution can also be clearly witnessed with SEM images in a larger scale, as shown in Fig. 7(c–e). Owing to the further enhanced diffusion energy, the AH kept increasing ×1.48 times and as a result of the compact aggregation of Au adatoms, the LD decreased by 17%, whereas, the AD increased ×1.39 times in phase I. The AD increase with an increase temperature from 750 to 850 °C can be due to the phase transition from the Au mounds to the hexagonal NPs. In phase II at 950 °C, the AD further decreased 47.4% due to the enhanced surface diffusion as expected, clearly shown in Fig. 7(b,e). However, both the AH and LD decreased 2.82% and 3.03% respectively at 950 °C likely due to the nanoscale Au evaporation as discussed. Accordingly, the R_RMS_ and SAR initially increased with the growth of nano-crystals in vertical size, and went down due to the decreased size and density of the nano-crystals, as shown in Fig. S6 and Table SII. Similarly, the bright spot in the 2-D FFT power spectra radically shrunk into smaller ones with a hexagonal pattern, suggesting the formation of the Au nanocrystals in Fig. 6(a-3)–(c-3). The self-assembled nano-crystal formation can be also witnessed with the elementary analysis shown in Fig. 8. As clearly shown by phase map in Fig. 8(b), the Au (yellow) distributes among the NPs, which means the shape transition happened with the formation of the Au NPs. As a result, the Au can be only detected in the area with NPs, whereas, the Si evenly exists in the areas with/without NPs, as shown with line-profiles in Fig. 8(c). Similarly, the Si and C Kα peaks can be equally witnessed in the both locations, whereas, the Mα1 peak at 2.123 KeV only occurred in the area with NPs, as shown in Fig. 8(d–e). The Au NPs are equally evidenced with the pillars indicating higher concentration in 3-D side-views of the Au map, which matched the holes in the Si map, as shown in Fig. 8(f,g).

**Figure 9:**
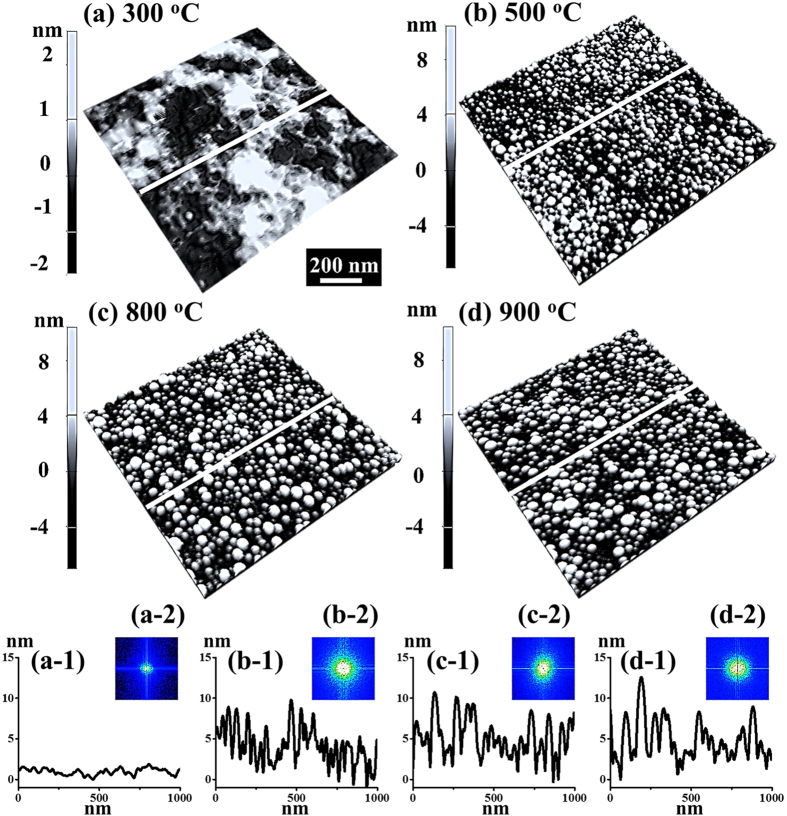
Evolution of the self-assembled Au nano-particles with a DA of 3 nm fabricated between 300 and 900 °C on 4H-SiC (0001). (**a**–**d**) AFM side-views 1 × 1 μm^2^. (**a-1**)–(**d-1**) Cross-sectional line-profiles acquired from the white lines in the corresponding AFM side-views. (**a-2**)–(**d-2**) 2-D FFT power spectra.

Figure 9 shows the evolution of the tiny dome-shaped Au NPs with a relatively low DA of 3 nm on 4H-SiC (0001) by the variation of AT between 300 and 900 °C. Figure S8 presents the corresponding samples with AFM top-views. Generally, with the increased AT, the tiny dome-shaped Au NPs were fabricated instantly after the annealing, and developed in size and density without the shape transition, which can be described with the Volmer-Weber growth model^26^,^27^. As mentioned, Au adatoms can only freely aggregated with a limited *l*_*D*_ in the pinholes perforated by the voids. Provided that the Au thin film with the DA of 3 nm is much thinner than 8 and 15 nm, the perforation can immediately happen at the initial stage with much less thermal energy. In addition, given that the bonding energy between Au atoms (*E*_*Au*_) was stronger than the bonding energy between Au adatoms and Si and C atoms (*E*_*I*_), namely, *E*_*Au*_ > *E*_*I*_, with the sufficient thermal energy supplied, Au adatoms can spontaneously nucleate to form the three-dimensional (3-D) islands (NPs). On the other hand, as discussed, the equilibrium shape can be decided by the surface energy for each orientation^41^,^42^, and with a small volume, the surface energy can still sustain isotropic, which can result in the spherical (dome) shaped Au NPs. As a result, the dome-shaped Au NPs were directly developed from the smooth Au thin film as soon as treated with sufficient thermal energy from 300 to 500 °C, as shown in Fig. 9(a,b). Accordingly, the surface modulation increased from ~1 nm to ~5 nm, as shown with line-profiles in Fig. 9(a-1),(b-1). Meanwhile, being providing with *E*_*Au*_ > *E*_*I*_, at elevated temperatures, the Au islands with larger boundaries tend to absorb more surrounding adatoms and merge smaller ones to form bigger in order to minimize the surface energy. Therefore, between 500 and 900 °C, with the enhanced *l*_*D*_, the dome-shaped Au NPs gradually increased in size at a expense of the Au NPs density, as shown in Fig. 9(c,d), which resulted in the slight increase of the surface modulation indicated by the line-profiles in Fig. 9(c-1),(d-1). As shown in Fig. S8(b)–S8(d), throughout the whole evolution, the tiny dome-shaped NPs were fabricated uniformly with a packed density at each AT, which results the symmetric bright spots instead of the irregular pattern in the 2-D FFT power spectra, as shown in Fig. 9(b-2)–(d-2). The evolved surface morphology can also be witnessed with the increase of the R_RMS_: Initially, the R_RMS_ drastically increased from 0.7 to 2 nm owing to the Au NPs formation, and subsequently, the R_RMS_ gradually increased as a function of the AT, as summarized in Fig. S8(e) and Table SIII. In short, the dome-shaped can be fabricated above 500 °C, and with the increased AT, the size of Au NPs increased with decreased density, which can be a conventional behavior for metallic NPs on various substrate, such as Ag NPs on the GaN and sapphire^45^,^46^, Au and Ga NPs on the GaAs^47^,^48^, and Au NPs on the Si^49^. Figure S9 shows the EDS spectra of the sample with 3 and 15 nm DAs annealed at 800 °C. As shown in Fig. S9(a) and S9(b), the increased DA can be evidenced with nearly 5 times higher counts in the Mα1 (2.123 keV) peaks of Au than that of 3 nm DA, as similarly witnessed with the Lα1 (9.711 keV) peaks, shown in Fig. S9(a-2) and S9(b-2).

## Conclusions

In summary, the systematical investigation on the evolution of the self-assembled Au nanostructures on N-type 4H-SiC (0001) controlled by varying the annealing temperature (AT) between 300 and 950 °C was successfully demonstrate with various deposition amounts (DAs): 3, 8, 15 nm. At higher DAs (8 and 15 nm), with the increased AT, the drastic morphology evolution of Au nanostructures was observed into two phases: (I) Au nano-mounds, and (II) hexagonal Au nano-crystals. Below 700 °C, the Au nano-mounds gradually formed and developed in the pinholes perforated by the nucleated voids as a function of the AT, which was discussed as a dewetting process in conjunction with the DLA model. Meanwhile, the hillocks were fabricated at the lower AT range between 500 and 600 °C, caused by the thermal expansion of Au film. Above 750 °C, with a sufficient thermal energy, the hexagonal nano-crystals can be successfully fabricated on 4H-SiC (0001) as a result of anisotropic distribution of surface energy caused by the increased volume. Finally, the size of Au NPs started to decrease above 850 °C likely due to the nanoscale dependent evaporation of Au nanocrystals. For the samples with 3 nm DA, the tiny dome-shaped Au NPs were fabricated based on the Volmer-Weber growth model without the formation of the irregular nano-mounds. With a small volume, the distribution of surface energy of the Au NPs was still isotropic, which eventually resulted in the dome shape rather than the polyhedral shape.

## Methods

In this experiment, the annealing temperature (AT) effect was investigated with 3, 8 and 15 nm deposition amounts (DAs) by the variation of annealing temperature in the pulsed laser deposition (PLD) system. Epi-ready N-type 4H-SiC (0001) substrate was ~250 μm thick with an off-axis of ±0.1° from the Technology and Devices International (TDI, USA). Prior to the growth, samples were treated with a chemical cleaning in the hydrofluoric acid (49.0–51.0%) solution for 10 minutes and subsequently flushed with the deionization (DI) water for three times. For each growth, samples were mounted on an Inconel holder with indium solder for a good thermal conduction of samples and degassed at 700 °C for 30 min under a chamber vacuum below 1 × 10^−4^ Torr. After degassing, the surface was quite flat without any contaminants as confirmed by the morphological and optical characterizations in Fig. S1 and S2. Subsequently, 3, 8 and 15 nm-thick Au thin films were deposited on the sample respectively in a plasma lion-coater at a growth rate of 0.05 nm/s with the ionization current of 3 mA below the vacuum of 1 × 10^−1^ Torr. To systematically investigate the AT effect, with the fixed DA and annealing duration, samples were systematically annealed at various ATs of 300, 500, 600, 700, 750, 800, 900, 950 °C with a ramping rate of 2 °C/s by a halogen lamp. After reaching each target substrate temperature, the samples were dwelled there for 450 s to ensure the uniformity of the Au nanostructures, and all the annealing process was strictly controlled with a computer-operated recipes in the PLD system under 1 × 10^−4^ Torr. To minimize the effect of the Ostwald ripening^50^, the temperature was immediately quenched down to the ambient temperature after each growth. For the morphological characterization, an atomic force microscope (AFM) was utilized for smaller area scanning with the non-contact (tapping) mode. AFM tips (NSC16/AIBS, μmasch) employed were with a radius of less than 10 nm curvature, made of Si etching. The tips were 17–21 μm long with the spring constant of ~42 N/m and resonant frequency of ~330 kHz. The cantilevers of the tips were back-side coated with ~30 nm Al to enhance the laser reflection. The same batch of tips were hired in order to diminish the tip effect for the consistency of the analysis and characterization. A scanning electron microcopy (SEM) was employed for larger area scanning. The acquired original data was processed and analyzed with the XEI software (Park Systems) to generate the AFM images, cross-sectional line-profiles, Fourier filter transform (FFT) power spectra, root-mean-squared (RMS) roughness, size and density plots of the Au NPs. The FFT power spectrum was obtained by converting the height distribution from the spatial domain to the frequency domain by the Fourier filter transform. Additionally, the energy-dispersive X-ray spectroscopy (EDS) with the spectral mode (Thermo Fisher Noran System 7) was employed for the elemental analysis and phase mappings under vacuum. For the optical characterization, the Raman spectrum was excited by a CW diode-pumped solid-state (DPPS) laser of a wavelength of 532 ± 1 nm with an output power of 120 mW and was received with a TE cooled CCD detector.

## Additional Information

**How to cite this article**: Li, M.-Y. *et al.* From the Au nano-clusters to the nanoparticles on 4H-SiC (0001). *Sci. Rep.* **5**, 13954; doi: 10.1038/srep13954 (2015).

## Supplementary Material

Supplementary Information
