# Corrigendum: From the Au nano-clusters to the nanoparticles on 4H-SiC (0001)

**DOI:** 10.1038/srep24470

**Published:** 2016-05-16

**Authors:** Ming-Yu Li, Quanzhen Zhang, Puran Pandey, Mao Sui, Eun-Soo Kim, Jihoon Lee

Scientific Reports 5: Article number: 13954;10.1038/srep13954 Published online: 09 10 2015; Updated: 05 16 2016

In the original version of this Article, the deposition thickness of Au ‘10 nm’ was incorrectly written as ‘8 nm’. As a result,

In the Abstract,

“With the relatively high DAs (8 and 15 nm), depending on the AT variation, the surface morphology drastically evolve in two distinctive phases, i.e. (I) irregular nano-mounds and (II) hexagonal nano-crystals.”

now reads:

“With the relatively high DAs (10 and 15 nm), depending on the AT variation, the surface morphology drastically evolve in two distinctive phases, i.e. (I) irregular nano-mounds and (II) hexagonal nano-crystals.”

In the legend of Figure 2,

“Evolution of the self-assembled Au nano-mounds on 4H-SiC (0001) at various annealing temperatures (AT) between 500 and 700 °C with 8 nm of Au deposition.”

now reads:

“Evolution of the self-assembled Au nano-mounds on 4H-SiC (0001) at various annealing temperatures (AT) between 500 and 700 °C with 10 nm of Au deposition.”

In the Results and Discussion section,

“In specific, the surface morphology with the 8 nm-thick Au deposition appeared quite smooth with only a few of nanometers of surface modulation, as shown in Fig. 2(a),(a-1).”

now reads:

“In specific, the surface morphology with the 10 nm-thick Au deposition appeared quite smooth with only a few of nanometers of surface modulation, as shown in Fig. 2(a),(a-1).”

“Similar to the 8 nm Au deposition, Au adatoms gradually aggregated and developed into the isolated irregular nano-mounds with the incremental variation of AT.”

now reads:

“Similar to the 10 nm Au deposition, Au adatoms gradually aggregated and developed into the isolated irregular nano-mounds with the incremental variation of AT.”

“Provided that the Au thin film with the DA of 3 nm is much thinner than 8 and 15 nm, the perforation can immediately happen at the initial stage with much less thermal energy.”

now reads:

“Provided that the Au thin film with the DA of 3 nm is much thinner than 10 and 15 nm, the perforation can immediately happen at the initial stage with much less thermal energy.”

In the legend of Figure 5,

“(**a**) SEM image of the sample with the 8 nm DA annealed at 600 °C.”

now reads:

“(**a**) SEM image of the sample with the 10 nm DA annealed at 600 °C.”

In the Conclusions section,

“In summary, the systematical investigation on the evolution of the self-assembled Au nanostructures on N-type 4H-SiC (0001) controlled by varying the annealing temperature (AT) between 300 and 950 °C was successfully demonstrate with various deposition amounts (DAs): 3, 8, 15 nm. At higher DAs (8 and 15 nm), with the increased AT, the drastic morphology evolution of Au nanostructures was observed into two phases: (I) Au nano-mounds, and (II) hexagonal Au nano-crystals.”

now reads:

“In summary, the systematical investigation on the evolution of the self-assembled Au nanostructures on N-type 4H-SiC (0001) controlled by varying the annealing temperature (AT) between 300 and 950 °C was successfully demonstrate with various deposition amounts (DAs): 3, 10, 15 nm. At higher DAs (10 and 15 nm), with the increased AT, the drastic morphology evolution of Au nanostructures was observed into two phases: (I) Au nano-mounds, and (II) hexagonal Au nano-crystals.”

In the Methods section,

“In this experiment, the annealing temperature (AT) effect was investigated with 3, 8 and 15 nm deposition amounts (DAs) by the variation of annealing temperature in the pulsed laser deposition (PLD) system.”

now reads:

“In this experiment, the annealing temperature (AT) effect was investigated with 3, 10 and 15 nm deposition amounts (DAs) by the variation of annealing temperature in the pulsed laser deposition (PLD) system.”

“Subsequently, 3, 8 and 15 nm-thick Au thin films were deposited on the sample respectively in a plasma lion-coater at a growth rate of 0.05 nm/s with the ionization current of 3 mA below the vacuum of 1 × 10^−1^ Torr.”

now reads:

“Subsequently, 3, 10 and 15 nm-thick Au thin films were deposited on the sample respectively in a plasma lion-coater at a growth rate of 0.05 nm/s with the ionization current of 3 mA below the vacuum of 1 × 10^−1^ Torr.”

These errors have now been corrected in the PDF and HTML versions of the Article, as well as the Supplementary Information file that now accompanies the Article.

