# Supplementary material for: From the Au nano-clusters to the nanoparticles on 4H-SiC (0001)
Source: Sci Rep. 2015 Sep 10;5:13954. doi: 10.1038/srep13954 (PMC4564804; doi:10.1038/srep13954)
Supplement: Supplementary Information [file srep13954-s1.doc]

Supplementary Information

**From the Au nano-clusters to the nanoparticles on 4H-SiC (0001)**

Ming-Yu Li^1^, Quanzhen Zhang^1^, Puran Pandey^1^, Mao Sui^1^, Eun-Soo Kim^1^ and Jihoon Lee^1,2*^

^1^ College of Electronics and Information, Kwangwoon University, Nowon-gu Seoul 139-701, South Korea ^2^ Institute of Nanoscale Science and Engineering, University of Arkansas, Fayetteville AR 72701, USA Correspondence and requests for materials should be addressed to J.L. ([jihoonleenano@gmail.com](mailto:jihoonleenano@gmail.com))

Financial support from the National Research Foundation (NRF) of Korea (no. 2011-0030821 and 2013R1A1A1007118), and in part by the research grant of Kwangwoon University in 2015 is gratefully acknowledged.


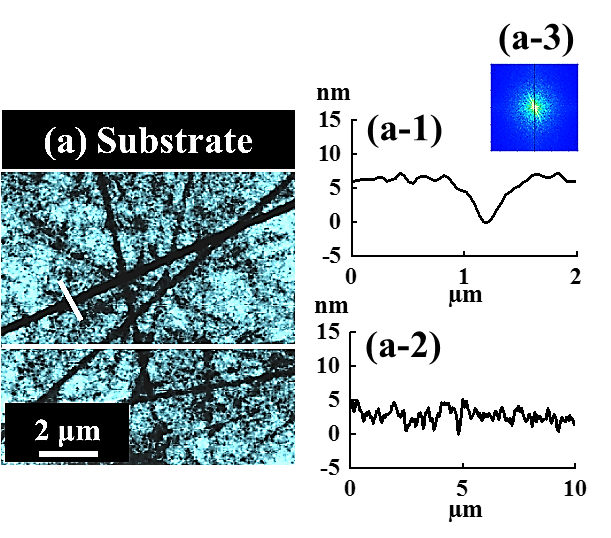


**Figure S1 | (a) AFM top-view of bare 4H-SiC (0001) substrate with an area of 10 × 10 μm^2^.** (a-1) Cross-sectional line-profile acquired from characteristic gaps in (a). (a-2) Line-profile from the 10 μm line. (a-3) 2-D FFT power spectra.


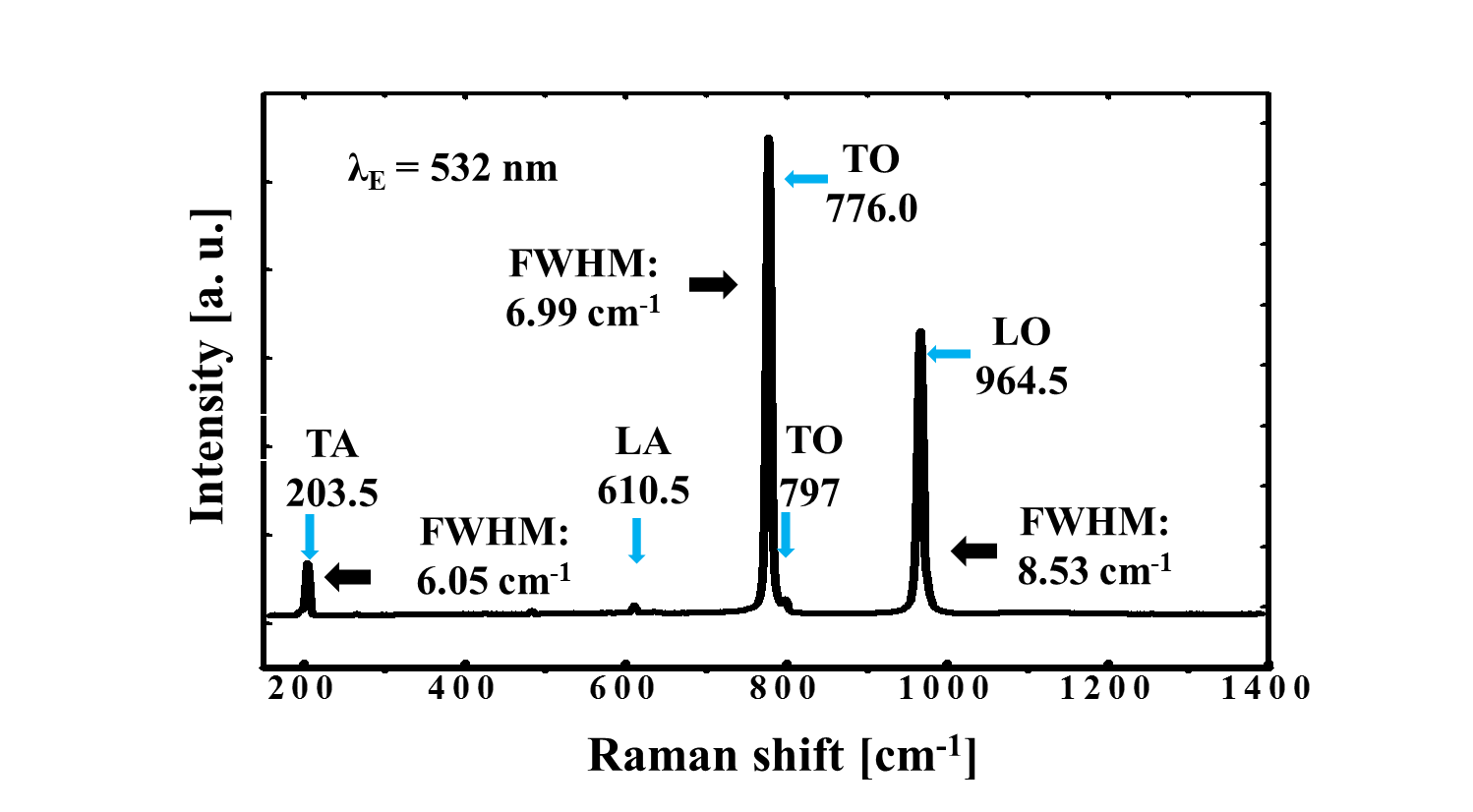


**Figure S2 | Raman spectrum (between 154 and 1388 cm^-1^) of 4H-SiC (0001) substrate at room temperature with a laser excitation of 532 ± 1 nm.** The peak of transverse acoustic (TA) was observed at 203.5 cm^-1^, and that of the longitudinal acoustic (LA) appeared at 610.5 cm^-1^. The two transverse optic (TO) peaks were at 776 and 964.5 cm^-1^, respectively. The longitudinal optical mode (LO) peak was shown at 964.5 cm^-1^.^1,2^ The full width at half maximum (FWHM) of the TA, TO and LO are 6.05, 6.99 and 8.53 cm^-1^, respectively.

1. Burton, J. C., Sun, L., Long, F. H., Feng, Z. C. & Ferguson, I. T. First-and second-order Raman scattering from semi-insulating 4 H-SiC. *Phys. Rev. B* **59**, 7282-7284 (1999).

2. Nakashima, S. & Harima, H. Raman Investigation of SiC Polytypes. *phys. stat. sol. A* **162**, 39-64 (1997).


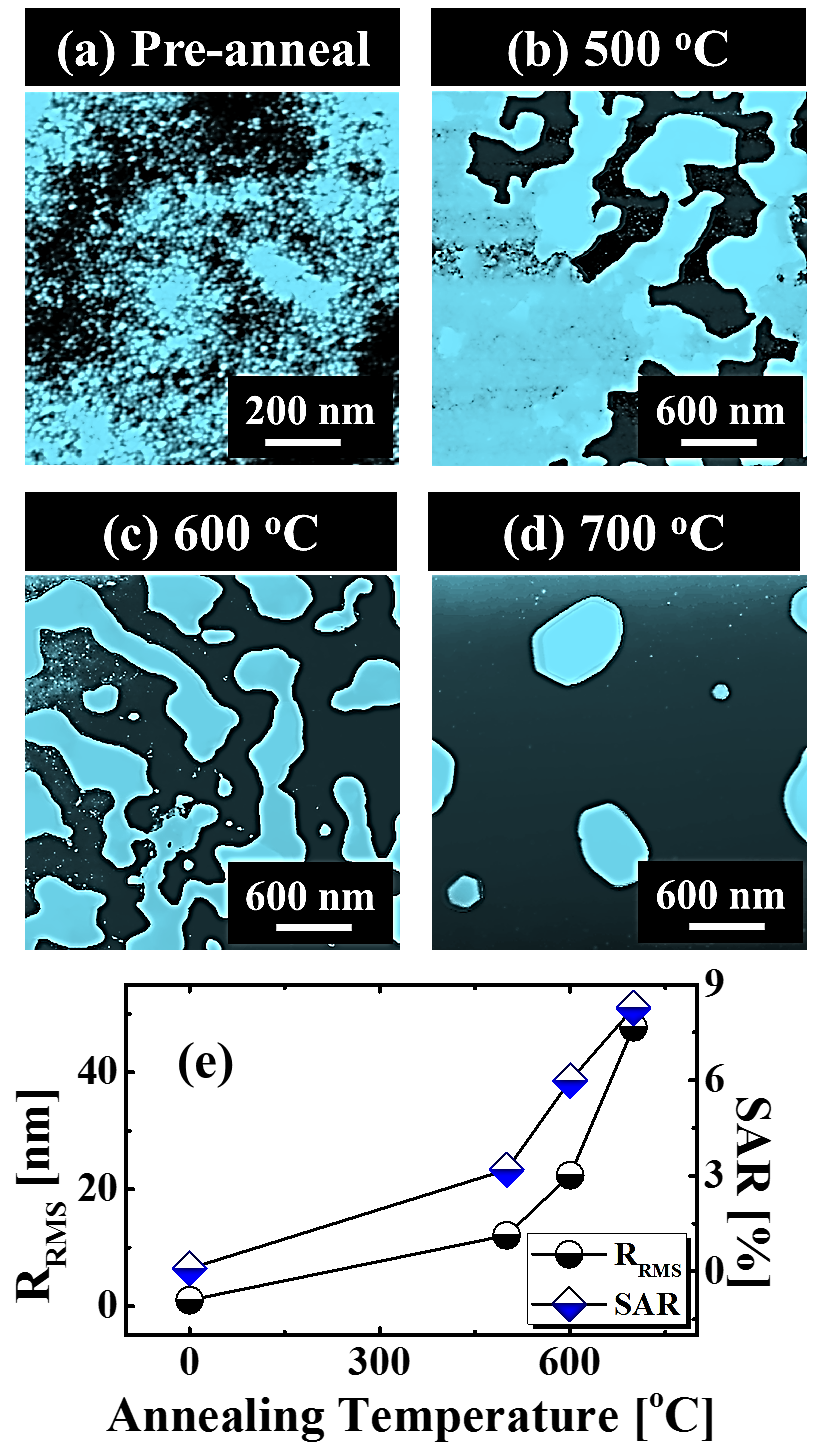


**Figure S3 | AFM top-views of surface morphology evolution by the variation of annealing temperature (AT) between 500 and 700 ºC with 8 nm of Au deposition annealed for 450 s.** (a) AFM top-views (1 × 1 µm^2^) of the pre-annealed surface. (b) – (d) AFM top-views of 3 × 3 µm^2^ at each ATs. (e) Summary of root-mean-squared roughness (R_RMS_) and surface area ratio (SAR). The SAR is given by
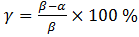
, in which [α] is the surface area (x × y) and [β] is the geometric area (x × y × z).


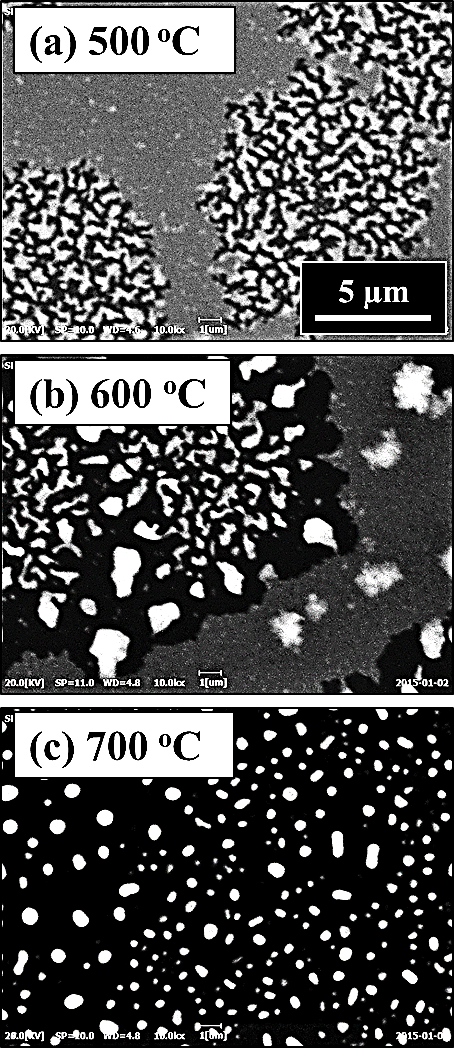


**Figure S4 | SEM images of self-assembled Au nanostructures by the variation of annealing temperature (a) 500, (b) 600 and (c) 700 ºC on 4H-SiC (0001).** The deposition amount and annealing duration were fixed at 8 nm for 450 s to clearly observe the annealing temperature effect. SEM images are 19.4 (x) × 14.6 (y) µm^2^.


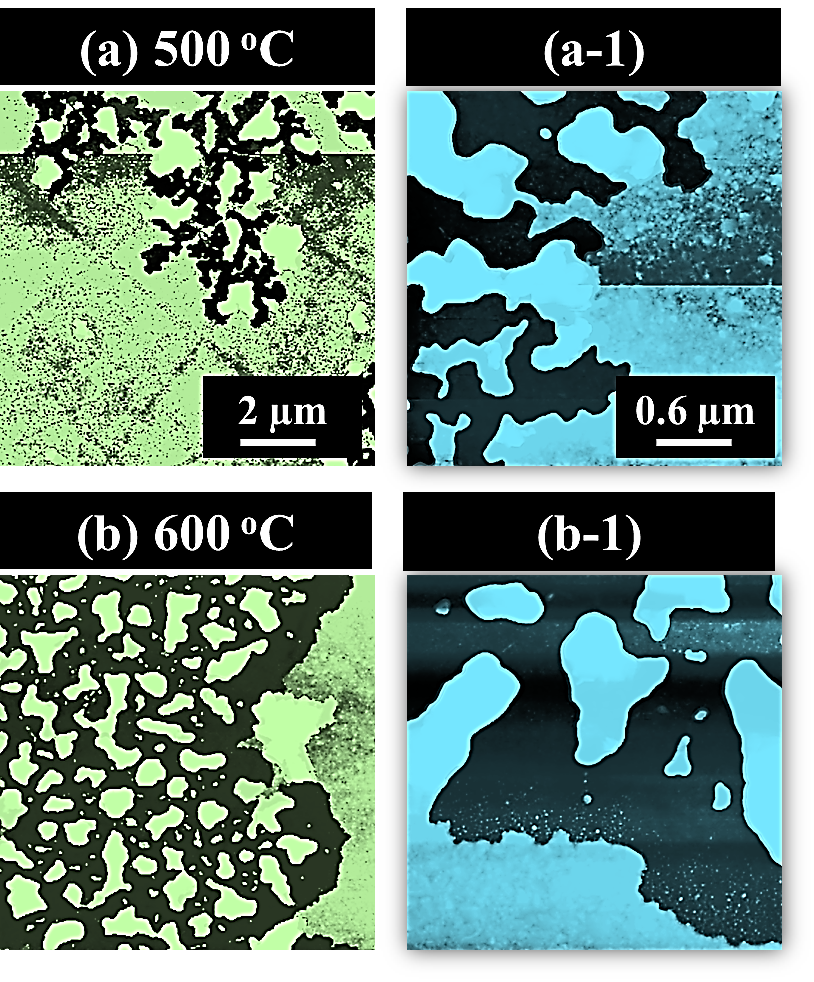


**Figure S5 | Surface morphology during the de-wetting process on 4H-SiC (0001) between 500 and 600 ºC with a DA of 15 nm.** (a) - (b) AFM top-views of 10 × 10 μm^2^. (a-1) – (b-1) 3 × 3 μm^2^.


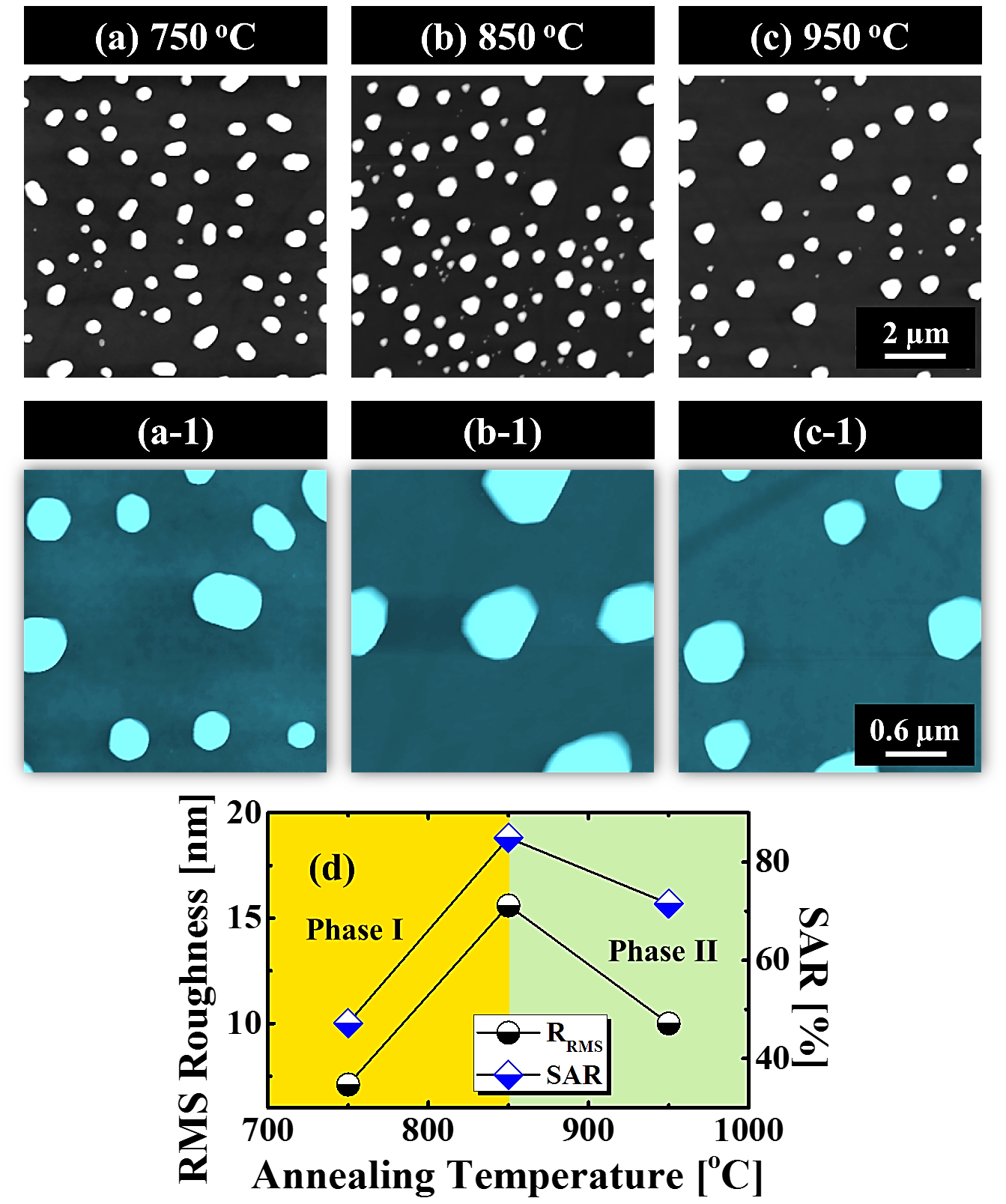


**Figure S6 | AFM top-views of the Au nano-crystals annealed between 750 and 950 ºC with the 15 nm DA on 4H-SiC (0001).** (a) - (c) AFM top-views of 10 × 10 μm^2^. (a) - (c) 3 × 3 μm^2^. (d) Plot of R_RMS_ and SAR.


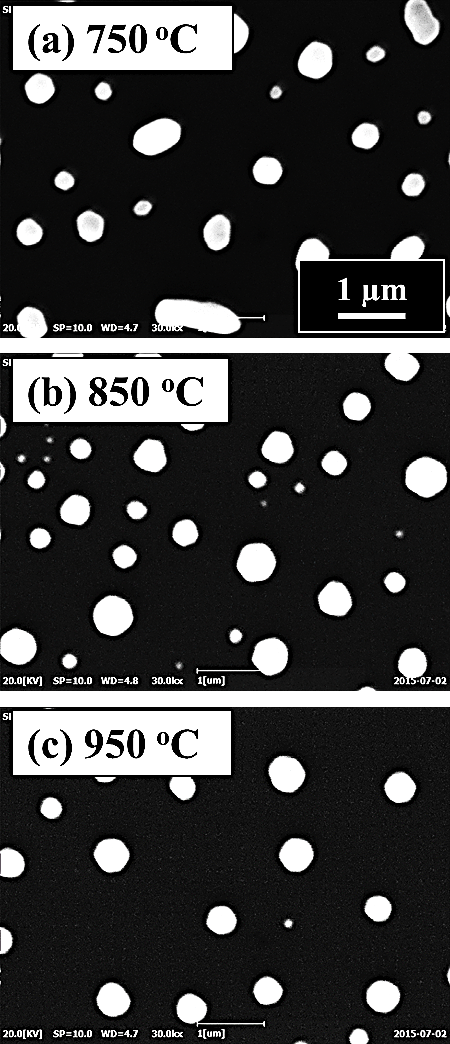


**Figure S7 | SEM images of self-assembled Au nano-crystals by the variation of annealing temperature (a) 750, (b) 850 and (c) 950 ºC on 4H-SiC (0001).** The deposition amount and annealing duration were fixed at 15 nm for 450 s to clearly observe the annealing temperature effect. SEM images are 6.7 (x) × 5.1 (y) µm^2^.


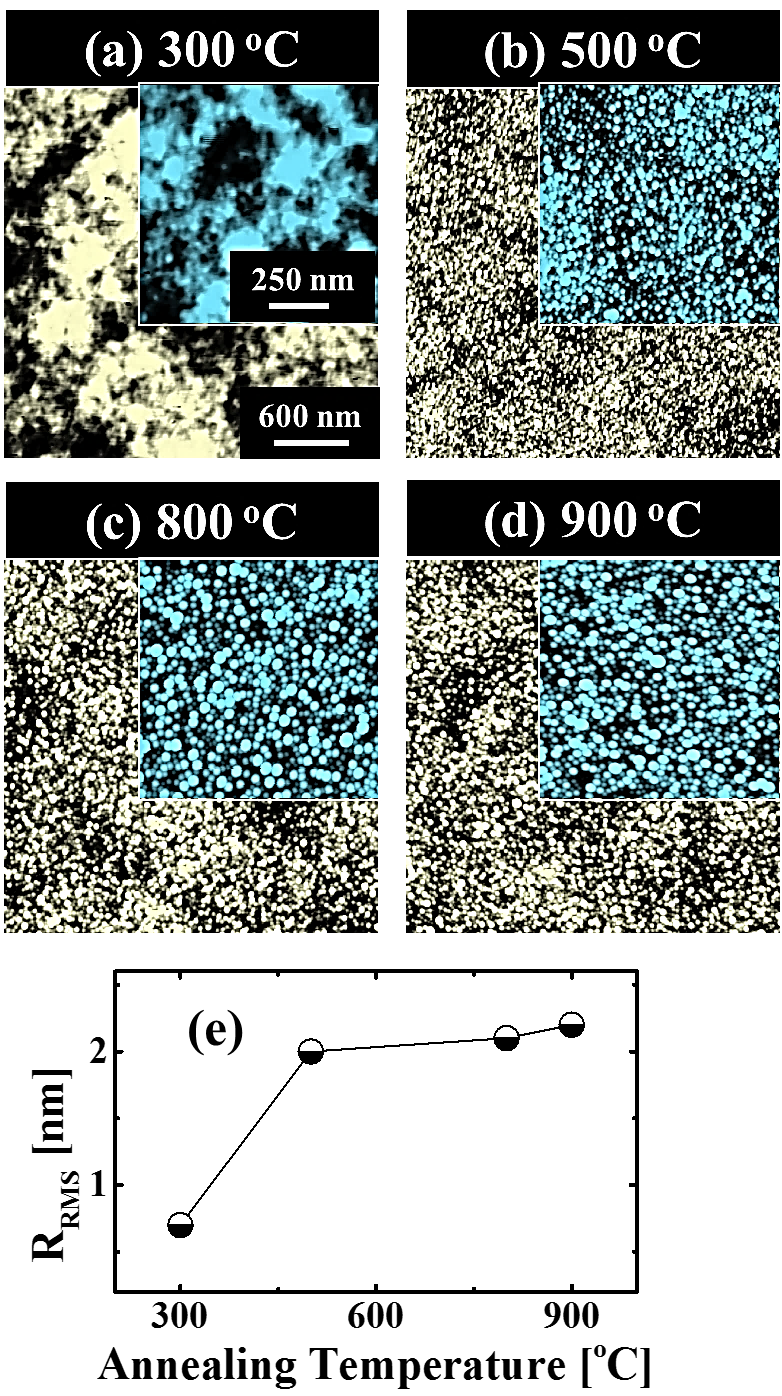


**Figure S8 | AFM top-views of dome-shaped Au nano-particles with a DA of 3 nm annealed between 300 and 900 ºC.** (a) - (d) AFM top-views of 3 × 3 µm^2^. (Insets) 1 × 1 µm^2^. (e) Plot of the R_RMS_ induced by the surface morphology evolution.


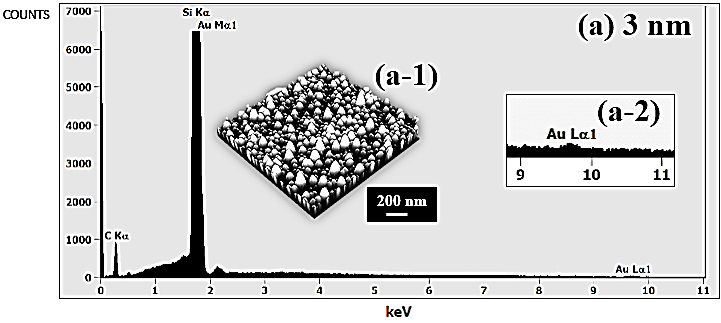

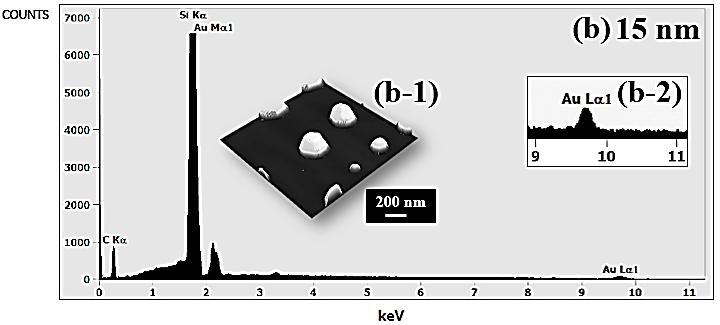


**Figure S9 | EDS spectra of the samples with the DA of 3 and 15 nm annealed at the equal condition at 800 ºC for 450 s on 4H-SiC (0001).** (a-1) & (b-1) AFM side-views of the corresponding samples. (a-2) - (b-2) Enlarged spectrum at range between 9 - 11 keV.

**Table SI | Summary of the R_RMS_, and SAR as a function of annealing temperature (AT) between 500 and 700 ˚C.** The DA was 8 nm.

| **AT**  **[˚C]** | **R_RMS_**  **[nm]** | **SAR**  **[%]** |
| --- | --- | --- |
| **0** | 1 | 0.09 |
| **500** | 12.1 | 3.19 |
| **600** | 22.4 | 5.99 |
| **700** | 47.7 | 8.27 |

**Table SII | Summary of the AH, LD, AD, R_RMS_, and SAR as a function of annealing temperature (AT) between 750 and 950 ˚C.** Each samples was deposited with a fixed DA of 15 nm. Error range: within ± 5 % for the AH, LD and AD.

| **AT**  **[˚C]** | **AH**  **[nm]** | **LD**  **[nm]** | **AD**  **[×10^7^ /cm^2^]** | **R_RMS_**  **[nm]** | **SAR**  **[%]** |
| --- | --- | --- | --- | --- | --- |
| **750** | 143.5 | 842.5 | 5.6 | 7.1 | 47.2 |
| **850** | 212.7 | 697.5 | 7.8 | 15.6 | 84.9 |
| **950** | 206.7 | 676.3 | 4.1 | 10 | 71.5 |

**Table SIII | Summary of R_RMS_ as a function of annealing temperature (AT) between 300 and 900 ˚C.** The Au nano-particles were fabricated with a fixed DA of 3 nm.

| **AT**  **[˚C]** | **RMS Roughness**  **[nm]** |
| --- | --- |
| **300** | 0.7 |
| **500** | 2.0 |
| **800** | 2.1 |
| **900** | 2.2 |
